# Psychometric Properties of a 36-Item Version of the “Stress Management Competency Indicator Tool”

**DOI:** 10.3390/ijerph13111086

**Published:** 2016-11-07

**Authors:** Stefano Toderi, Guido Sarchielli

**Affiliations:** 1 Department of Psychology, University of Bologna, Viale Berti Pichat, 5-40126 Bologna, Italy; 2 School of Psychology and Education, University of Bologna, Piazza Aldo Moro, 90-47521 Cesena (FC), Italy; guido.sarchielli@unibo.it

**Keywords:** work related stress, Stress Management Competency Indicator Tool, supervisors’ behaviours, psychometric properties, Stress Management Indicator Tool, Management Standards

## Abstract

The development of supervisors’ behaviours has been proposed as an innovative approach for the reduction of employees’ work stress. The UK Health and Safety Executive (HSE) developed the “Stress Management Competency Indicator Tool” (SMCIT), designed to be used within a learning and development intervention. However, its psychometric properties have never been evaluated, and the length of the questionnaire (66 items) limits its practical applicability. We developed a brief 36-item version of the questionnaire, assessed its psychometric properties and studied the relationship with the employees’ psychosocial work environment. 353 employees filled in the brief SMCIT and the “Stress Management Indicator Tool”. The latter is a self-report questionnaire developed by the UK HSE, measuring workers’ perceptions of seven dimensions of the psychosocial work environment that if not properly managed can lead to harm. Data were analysed with structural equation modelling and multiple regressions. The results confirmed the factorial structure of the brief SMCIT questionnaire and mainly supported the convergent validity and internal consistency of the scales. Furthermore, with few exceptions, the relations hypothesized between supervisors’ competencies and the psychosocial work environment were confirmed, supporting the criterion validity of the revised questionnaire and the UK HSE framework. We conclude that the brief 36-item version of the SMCIT represents an important step toward the development of interventions directed at supervisors and we discuss the practical implications for work stress prevention.

## 1. Introduction

Psychosocial factors of the work environment, such as job demands, control, and social support, if not properly managed can be the cause of work stress for employees. This is a well-known phenomenon of the modern work environment and is responsible for serious negative consequences for the individual, the organization, and society in general.

Theoretical frameworks on work stress have been developed since the seminal works of Hans Selye and, nowadays, several perspectives are available, such as the Job Strain Model [1], the Effort-Reward Imbalance Model [2], and the Job Demands-Resource Model [3]. On the other hand, practical interventions aimed to reduce work stress have mainly been developed only in the last two decades, and their efficacy is still under consideration by researchers.

Interventions can be broadly divided into an individual and organizational level [4]. Organizational interventions (i.e., science-based actions that target a relatively large number of individuals [5]) aim to change the organizational and psychosocial environment, providing a more preventative strategy (primary prevention). Thus, organizational interventions have been widely recommended [6]. However, Cox et al. [4] pointed out that available organizational interventions often fail to achieve the desired results, and, therefore, it is necessary to focus on truly innovative approaches. One of these has been identified by both academic [4,5,6,7] and institutional [8] publications in the development of leadership or supervisors’ behaviours. In fact, it has been noted that supervisors are key figures in different phases of the work stress process [9,10], and they can significantly influence the efficacy of interventions directed to employees.

One of the ways in which supervisors can affect employees’ level of stress is through a crossover contagion process [11]: supervisors can transmit their level of stress and poor affective well-being to employees simply by interacting with them. Moreover, supervisors’ behaviours (e.g., support, acting with integrity, etc.) have been found to directly influence a large variety of employees’ outcomes, such as mental health strain [12], job satisfaction [13], psychophysiological outcomes [14], early retirement, and work-related disability [15].

At the same time, given their role and position, supervisors organize and manage the employees’ work (e.g., planning and monitoring employees’ workload, delegating job assignments, assigning autonomy), influencing job characteristics (e.g., role, autonomy, demands) and determining in part the employees’ psychosocial work environment. As a consequence, supervisors can influence employees’ work stress or well-being by impacting on the presence/absence of psychosocial hazards, which suggests that they should monitor and improve their own behaviours [16].

Finally, supervisors have a critical role in the implementation of interventions aimed to reduce employees’ work stress. Different studies found that supervisors can facilitate interventions by assuming an active role [17], providing feedback [18] and supportive behaviours to employees [19], and developing action plans [18]. On the contrary, supervisors may inhibit the psychosocial work environment changes if they do not have proper awareness of the stressful job effects on workers, do not feel equipped to deal with work stress, or perceive this issue as irrelevant to their staff [20]. This last point is particularly important given that leaders’ health has been found to be related to their evaluation of the level of employees’ stress [21,22]. In summary, supervisors can either “make or break” an intervention and have been identified as the “drivers of change” [23].

These research findings provide strong support for the development of intervention activities targeting the supervisors and for their involvement in work stress programmes. However, such activities and involvement are still scarce in the literature and practice.

One notable exception is provided by the UK agency Health and Safety Executive (HSE), which developed a management competencies framework for preventing and reducing stress at work [24,25]. It is based on the idea, described above, that supervisors’ behaviours have an effect on employees’ psychosocial working environment. The HSE proposed the framework in the context of its activities on the Management Standard method for work stress prevention. This approach [26] identified seven dimensions of the psychosocial work environment (demands, control, managers’ support, peers’ support, role, relationship, and change) that if not properly managed can lead to harm, and proposed a set of “Management Standards” that should be achieved by the organization to prevent work stress. The performance of the organization against the Standards (i.e., workers’ perceptions of the seven dimensions of the psychosocial work environment) is measured through a self-report questionnaire, the UK HSE “Stress Management Indicator Tool”.

The framework developed is designed to be used within a learning and development intervention, where supervisors’ positive and negative behaviours are measured in order to aid supervisors in assessing and improving their work stress management competencies. In particular, the intervention, after the measurement of the supervisors’ behaviours by both subordinates and the supervisor him/herself, is divided into two phases: (a) an upward feedback report given to the supervisor, showing how his/her behaviours were perceived by subordinates and allowing comparison between the self/others scores; (b) a workshop directed to supervisors aiming to explore the importance of supervisor behaviours, increase awareness of their own behaviours, and help them to foster positive behaviours.

This approach has various positive strengths: (a) it is based on a management competencies framework and questionnaire (the so-called Stress Management Competency Indicator Tool—SMCIT [5]) specifically developed for the well-being and work stress issue [24]. This is a positive characteristic because questionnaires drawn from a priori models of leadership may fail to fully capture the specific behaviours relevant for work and well-being management [24]; (b) it was suggested as an intervention inside the HSE Management Standard method [26] described above, one of the most advanced in Europe for work stress evaluation and prevention [27]. Indeed, the intervention (and the SMCIT questionnaire) is theoretically linked to the psychosocial hazards identified by the HSE method (i.e., demands, control, social support, role, change, and relationship) and could be more strictly linked to the evaluation phase; (c) there is evidence of the efficacy of such an intervention [28], and Eurofound and the European Agency for Safety and Health at Work (EU-OSHA) [8] presented it and the SMCIT as good practice for the development of positive leadership and supervisor behaviours, thus stimulating its diffusion and application.

On the other hand, almost one decade after its development, no empirical research about the framework has been published; the only exception being Lewis et al. [29] (who used qualitative data derived from phase 1 [24]). Toderi et al. [30] proposed that the main limitation of the approach can be identified in the SMCIT questionnaire itself, which is the basis of all the following phases of the intervention. In fact, the SMCIT contains a high number of items (66), making its use difficult for interventions and research, especially if further variables are added. Moreover, its factorial structure has never been assessed, and the only data available [25] referred to a statistical reduction of a previous 112-item version. Finally, data on the reliability of the scales and how the scales correlate with other relevant variables are not available to date.

The general aim of this study is, therefore, to revise the SMCIT questionnaire and overcome these limits by proposing a shorter 36-item version, evaluating its psychometric properties and their associations with psychosocial factors.

In the next section, we briefly describe the structure and previous development of the SMCIT, identifying what is still in need of improvement. We then describe the specific aims of the study and how they contribute to the advancement of research and practice on this topic.

### 1.1. The Stress Management Competency Indicator Tool and Its Development

The SMCIT is a questionnaire developed in two research phases [24,25] of the UK HSE project “Management Competencies Framework for Preventing and Reducing Stress at Work”. In the first phase, 19 supervisors’ competencies associated with the effective management of work stress were identified on the basis of a literature review and a qualitative study [24]. In the second phase [25], the number of behavioural competencies was reduced in order to make the framework usable in practice and to develop a questionnaire for the measurement of supervisors’ behaviours. The result was a 112-item questionnaire, administered successively to 656 workers at 22 organizations. Exploratory factor analysis revealed a four-factor solution (competencies) of the questionnaire and items with problematic factor loadings were deleted. The final result consists in the 66-item version of the SMCIT, further explored in two workshops of stress experts (*n* = 38), naming each factor (competency) and identifying sub-clusters (sub-competencies). In the same workshops the experts hypothesized that each supervisor’s competency has an impact on specific Management Standards (as measured by the “Stress Management Indicator Tool”) and these associations were established. The final framework of the SMCIT questionnaire is summarized in Table 1, where the last column also reports the 36 items of the brief questionnaire proposed in this study.

As can be seen in Table 1, the first competency is named *Respectful and Responsible* (RR) and describes the behaviours of a supervisor who shows integrity and is able in managing emotions. It is measured by 17 items of the 66-item questionnaire and includes the subdimensions of integrity (respectful and honest to employees), managing emotions (behaves consistently and calmly), and considerate approach (thoughtful in managing others and delegating). These behaviours are expected to have a great impact on the employees’ work environment, influencing all the psychosocial factors except role and change.

The second competency, named *Managing and Communicating existing and future Work* (MCW), is measured by 22 items and includes the subdimensions of proactive work management (monitors existing work, allowing future prioritization and planning), problem solving (deals with problems promptly, rationally and responsibly), and participative (listens and consults with team, provides direction, autonomy and opportunities). These behaviours are expected to impact demands, control, support and role.

The third competency, named *Reasoning/Managing Difficult Situations* (RDS), is measured by 12 items and includes the subdimensions of managing conflict (deals with conflicts fairly and promptly), use of organizational resources (seeks advice when necessary from managers, human resources, occupational health), and taking responsibility for resolving issues (supportive and responsible approach to issues). These behaviours are expected to impact only support and relationships.

The fourth competency, named *Managing the Individual within the Team* (MIT), is measured by 15 items and includes the subdimensions personally accessible (available to talk to personally), sociable (relaxed approach, such as socializing and using humour), and empathetic engagement (seeks to understand workers in terms of their motivation, point of view, and life). These behaviours are expected to impact control, support and relationships.

As explained previously, the length of the questionnaire and the lack of evidence regarding its psychometric properties actually limit its applicability. In order to overcome these limits, Toderi et al. [30] focused on one out of four competencies of the SMCIT (*Managing and Communicating existing and future Work*) and proposed a brief 9-item version of the scale (instead of the original 22 items). Their results from 178 employees of two Italian public organizations revealed excellent psychometric properties of the supervisors’ behaviour scale and confirmed the expected relationships with criterion outcomes. In particular, positive supervisor behaviours in *Managing and Communicating existing and future Work* were associated with lower employee anxiety and greater enthusiasm and team effectiveness. However, these results were limited to one competency only of the framework.

### 1.2. Aims of the Study

Given the results obtained by Toderi et al. [30] and the positive advantage of a brief tool, we aim to further develop the SMCIT questionnaire in two ways: (a) developing a full brief version of the tool (i.e., measuring all four competencies of the HSE framework) and evaluating its psychometric properties; and (b) studying the relationship between the four competencies measured and the employees’ psychosocial factors of the working environment.

Regarding the first goal, the high number of items of the SMCIT (66) makes it difficult and expensive to use in practice. Furthermore, these kind of interventions (i.e., supervisors’ evaluation) usually involve a relatively low number of participants, making any psychometric evaluations of long questionnaires such as the 66-item SMCIT difficult to perform.

On the other hand, the benefits linked to short, valid, and reliable diagnostic tools are numerous [31]: they take less time to complete, there are fewer missing data, and there is a higher response rate. Moreover, shorter measures of variables make it easier to include other measurements in the questionnaire (e.g., psychosocial factors, strain measures, etc.), which permit a more comprehensive data collection for intervention and research purposes. Overall, a brief, valid and reliable version of the questionnaire will permit easier use in practice and also allow for a psychometric evaluation of the tool and the measurement of other relevant constructs.

Following the procedure of Toderi et al., we developed a 36-item version of the SMCIT (using nine items for each of the four competencies, three items for each sub-competency) and aimed to evaluate its psychometric properties (i.e., factorial structure, validity and reliability) within a confirmatory framework.

The second goal of the research is to study the relationships between supervisors’ behaviours and employees’ psychosocial factors of the working environment. An empirical evaluation of these relationships (i.e., showing if and how supervisors’ management competencies determine the employees’ work environment) could provide criterion validity to the questionnaire, also supporting the theoretical framework developed. Furthermore, this kind of knowledge has practical implications by showing which psychological factors could be improved through the development of specific supervisors’ management competencies. As reported above (see also the second column of Table 1), 38 stress experts hypothesized the relationships expected: the competency *Respectful and Responsible* (RR) was hypothesized to influence demands, control, social support, and relationship; the competency *Managing and Communicating existing and future Work* (MCW) was hypothesized to influence demands, control, social support, and role; the competency *Reasoning/Managing Difficult Situations* (RDS) was hypothesized to influence social support and relationships; the competency *Managing the Individual within the Team* (MIT) was hypothesized to influence control, support and relationships. None of the four competencies was directly linked to the Management Standard areas of “Change”.

These hypothesized relationships have never been evaluated, so our second aim is to study if they can be empirically confirmed.

## 2. Methods

### 2.1. Procedure and Participants

The research was conducted in 26 organizations that accepted to participate in a study aimed to improve supervisor’s competencies as a work stress prevention activity. A total of 39 work teams (i.e., a group composed of a supervisor and at least 10 members) were identified in collaboration with the HR offices and the supervisors were invited to participate in the research activities. Their subordinates were also invited to participate (*n* = 582) and to fill in anonymously a structured self-administered questionnaire. Three hundred and three workers returned the questionnaire filled in correctly (60.6% response rate). One hundred and ninety-two were men (54.4%), 16.1% aged less than 29, 29.7% between 30 and 39, 34.8% between 40 and 49, and 14.2% aged 50 or more. The mean working experience was 17.43 years (SD = 10.43) and the mean tenure in the team was 8.48 years (SD = 7.30).

The investigation was conducted between January and April 2016 and the study protocol was approved by the Bioethics Committee of the University of Bologna, with Code No.152-5/2/2016.

### 2.2. Scales Development and Measures

The 36-item short version of the SMCIT was built starting from the nine items of the MCW scale validated by Toderi et al. (2015), and developing the remaining 27 items (instead of the original 44) that measure the RR, RDS, and MIT competencies. Firstly, following the procedure adopted by Toderi et al. [30], the 44 items of the original full version were translated into Italian, through a back-translation procedure. Secondly, an items reduction was made by adopting the criterion that the four competencies should all have the same number of items (nine items each; three items for every sub-competency). In particular, for each subcompetency the description provided by Yarker et al. [25] and reported above was taken into account, and in the light of this, the three most representative items were selected. For example, the first sub-competency is “Integrity”, which defines a supervisor as “Respectful and honest to employees” [25]. The three items “Doesn’t speak about team members behind their backs“, “Is honest”, and “Treats me with respect” were retained as the most representative of the dimension by both the authors, and the other items were discarded (“Is a good role model”; ”Says one thing, then does something different”).

The final result of this process is the 36-item short version of the SMCIT shown in the last column of Table 1.

In accordance with the original version of the questionnaire, employees responded on a 5-point Likert scale ranging from 1 (strongly disagree) to 5 (strongly agree).

Psychosocial factors of the working environment (i.e., demands, control, manager and peer support, relationships, role, change) were measured through the UK HSE Stress Management Indicator Tool [32,33] as developed in a short 25-item version [34,35] and validated in Italian by Balducci et al. [36]. Each item is rated on a 5-point Likert scale, with possible responses ranging from never to always or from strongly disagree to strongly agree, according to the specific items. Higher scores on all the psychosocial factors indicate a better psychosocial environment. Thus, for example, a high score on the demands scale corresponds to low job demands.

### 2.3. Analyses

The data were analysed using the Software IBM SPSS Statistics 23 (IBM SPSS Inc., Chicago, IL, USA) and IBM SPSS AMOS 23 (IBM SPSS Inc., Chicago, IL, USA).

First, correlational analyses to explore the relationships between supervisor’s competencies and psychosocial factors were conducted.

Secondly, the psychometric properties of the 36-item SMCIT questionnaire were studied using a confirmatory approach. To confirm the proposed factorial structure of the questionnaire (i.e., four supervisors’ competencies) we compared two alternative measurement models (estimation method maximum likelihood): the hypothesized four-factor model against a one-factor model (in which all items loaded on one factor). The results were evaluated by using the χ^2^ statistic and the most suggested indices in the literature [37,38]: comparative fit index (CFI), with values 0.90 or higher indicating acceptable fit [39]; the root mean square error of approximation (RMSEA), with values of 0.08 or lower indicating acceptable fit [40]; and the standardized root mean square residual (SRMR), with values of 0.10 or lower indicating acceptable fit. Furthermore, convergent validity (the extent to which two measures of the same concept are correlated) was studied through standardized lambda parameters and the average variance extracted (AVE) [41]. The former represents the regression coefficients relating each observed variable with the latent one; significant coefficients greater than 0.50 point out a strong condition of convergent validity. The latter is the amount of variance that is captured by the latent construct in relation to the amount of variance due to its measurement error; acceptable convergent validity is considered to be shown with a value of 0.50 or higher (i.e., the common variance of a set of items captured by the latent construct is more than 50%). Finally, internal consistency of the scales, the extent to which the items of a scale are measuring the same construct, was studied through both the Cronbach’s alpha and the Composite Reliability (CR) index. Values greater than 0.70 are recommended [42].

Thirdly, the relationship between supervisors’ competencies and psychosocial factors (criterion validity, i.e., the extent to which a measure is related to an outcome that it should theoretically be able to predict) was evaluated by conducting seven multiple regression analyses (one for each psychosocial factor) and introducing the four supervisors’ competencies together as independent variables in the first step. The results were evaluated considering the hypothesized relationships provided by Yarker et al. [25]. A confirmation of these relationships will provide support both to the framework developed by the HSE (i.e., the competencies selected are significant for the work stress issue) and to the validity of the questionnaire developed (i.e., the SMCIT significantly predicts psychosocial factors, which are outcomes theoretically linked to the supervisors’ competencies).

## 3. Results

### 3.1. Correlational Analyses

Table 2 shows the results of the correlational analyses, along with descriptive data (variable’s means and standard deviations) and internal consistencies (Cronbach’s alpha).

As expected, the four supervisors’ competencies are correlated positively to each other and show positive and highly significant correlations (*p* < 0.001) with all the seven psychosocial factors. Managerial support is the dimension showing the highest values (ranging from 0.73 to 0.82), while the correlation coefficients with the other psychosocial factors range between 0.24 and 0.68. The internal consistencies of the scales are all good or excellent (ranging from 0.78 to 0.92).

### 3.2. Psychometric Properties of the 36-Item SMCIT

Table 3 shows the results of the two confirmatory factor analyses. The proposed model (one, four factors) shows an acceptable fit to data, with RMSEA < 0.08, and CFI = 0.90. On the contrary, the 1-factor model shows a statistically significant deterioration in the χ^2^ (Δχ^2^_M2–M1_ (10) = 579.96, *p* < 0.001) and worst fit: the RMSEA rises to the cut-off value 0.08 and the CFI shows a non-acceptable index (0.83). Thus, the four factor (competencies) structure of the questionnaire best fit the data in an acceptable way.

**Figure 1:**
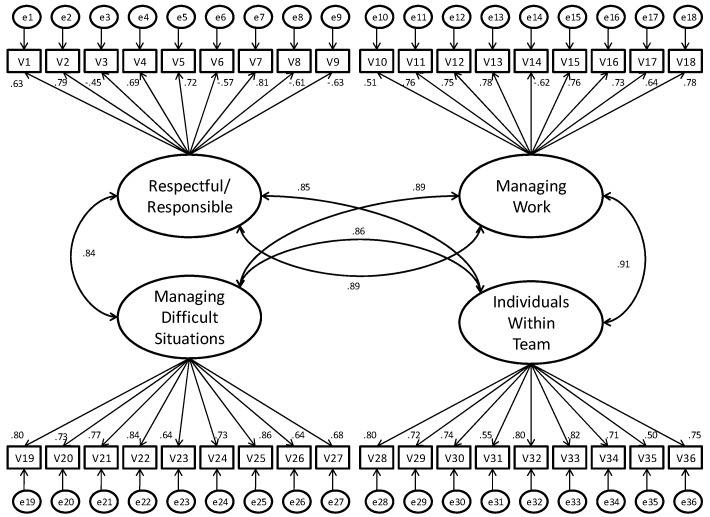
The Four-Factors proposed model and standardized path coefficients.

The standardized path coefficients of the four-factor model are shown in Figure 1. They are always significant and higher than 0.50, with the sole exception of item V3 of the RR scale (“Creates unrealistic deadlines for delivery of work”), which obtains a value of −0.45. In addition, AVE coefficients for the competencies MCW, RDS, and MIT are, respectively, 0.50, 0.56, and 0.52, satisfying the threshold (greater than 0.50) suggested by Fornell and Larcker [41]. However, the threshold is not fully satisfied for RR (0.44). Taken together, these results confirm the convergent validity of the four scales measurement model, even though there is some concern for the RR scale and item 3 in particular.

Finally, the reliability of the scales, evaluated through the Cronbach’s alpha above, was further explored using the CR index. The index is based on proportions of variance (lambda parameters) and takes account of each item’s error, providing a less biased estimate of reliability than Cronbach’s alpha [43]. The CR indexes for the competencies RR, MCW, RDS, and MIT are, respectively, 0.83, 0.82, 0.80, and 0.82, satisfying the threshold (greater than 0.70) and providing strong support to the reliability of the scales.

### 3.3. Criterion Validity and Relations with Psychosocial Factors

Table 4 reports the results of the multiple regressions conducted to evaluate criterion validity and the effects of the four supervisors’ competencies on psychosocial factors.

The results show that, taken together, the supervisors’ competencies explain a good part of the variance of all the psychosocial factors, ranging from 71% for managers’ support to 21% for demands. It should also be noted that even if the 38 stress experts did not directly link the four competencies to change, we found a significant effect of MCW (β = 0.49, *p* < 0.001) and RDS (β = 0.17, *p* < 0.05), and supervisors’ competencies globally explained 48% of variance. Thus, these results provide a first support to the relation existing between supervisors’ competencies and psychosocial factors.

Considering the predictive role of each competency, RR was predicted to affect demands, control, support, and relationship. All these relations are confirmed by the results. Splitting support into that of managers and peers, only the first is influenced by RR.

The competency MCW was predicted to affect demands, control, support, and role. Of these, only the effect of MCW on demands is not confirmed.

The competency RDS was predicted to affect social support and relationships and both are confirmed by the data.

The competency MIT was predicted to affect control, support, and relationships. Also, in this case all the relations are confirmed, but the MIT competency shows a counterintuitive negative relation with control and relationship. These results will be further discussed below.

Overall, the predictive role of supervisors’ competencies is confirmed by the results and the relationships hypothesized by the 38 stress experts are largely identified in our study.

## 4. Discussion

The UK HSE “Management Competencies for Preventing and Reducing Stress at Work” framework and the SMCIT questionnaire are considered innovative constructs and tools for work stress prevention [8]. However, the length of the questionnaire and the lack of empirical evidence actually limit its use in practice [30]. In this study, we aimed to develop a brief 36-item version of the questionnaire and evaluate its psychometric properties and relations with psychosocial factors.

Overall, our results provide support to the validity of the short version, confirming the adequacy of the four competencies structure of the questionnaire, its convergent validity, and internal consistency. The only exception is the competence *Respectful and Responsible*—managing emotions and having integrity”, which failed to provide sufficient convergent validity. The value of AVE was 0.44, suggesting that a substantial part of the variance in the items was explained by the measurement error, rather than by the underlying factor. An examination of the standardized path coefficients (see Figure 1) suggests that this result could be attributed to the subcompetency “Considerate approach” (thoughtful in managing others and delegating), measured by the items numbered 3, 6, and 9. All these items, in fact, showed low standardized path coefficients and, in particular, item 3 (“Creates unrealistic deadlines for delivery of work”) obtained a non-satisfactory value of −0.45. Indeed, if the three items are removed from the compute of the AVE index, the value rises to an acceptable 0.50. It should be noted that in the exploratory factor analyses presented by Yarker et al. [25], factor loadings for this subcompetency (measured by six items) were all low as well, with one value of 0.57 and all the others below 0.51. As a consequence, it seems that the convergent validity problem that we found in the first competence (Respectful/Responsible) might not be linked to the reduction in items, but already present in the full version of the SMCIT and ascribable solely to the sub-dimension “Considerate approach”. Consequently, future research should further investigate the psychometric properties of this scale in other samples, first substituting item 3 with other initially discarded items (e.g., “Makes short-term demands rather than allowing me to plan my work”). Alternatively, new items mapping the subcompetency content could be developed and evaluated.

It should be noted that even though we used the most suggested indexes for our confirmatory approach (i.e., CFI, RMSEA, and SRMR [37,38]) other useful fit indexes (e.g., Normed Fit Index and Adjusted Goodness of Fit Index) could be tested as well in future research, providing a deeper assessment of the questionnaire.

A second contribution provided by our results is related to the association existing between supervisors’ competencies and psychosocial factors. On the one side, these results confirm the supervisor’s role in creating healthy workplaces [12,16]. On the other, the results provide criterion validity to the brief 36-item SMCIT and support the “Management Competencies framework for Preventing and Reducing Stress at Work” developed by HSE. In fact, the different impact of specific competencies on specific psychosocial factors hypothesized in Yarker et al. [25] were largely supported in this study.

Some of our results merit further comment. Firstly, in our study the supervisors’ competencies explained 71% of the dimension managers’ support. Even if the variance is very high, we believe that this result is not surprising given that both refer to the behaviours of managers and all four supervisors’ competencies were predicted to influence the dimension support (see Table 1).

A second point concerns the “change” dimension of the working environment (i.e., how organisational change is managed and communicated in the organisation). The work stress experts suggested that although many management behaviours could be beneficial during times of change, none of them is specific to this context. However, our results showed that the communication of existing and future work (MCW) and the management of conflicts and difficult situations (RDS) significantly predict change. If confirmed by future research, this result could help to refine the framework, suggesting that supervisors’ competencies play a role in all the management standards.

A surprising exception to the positive results observed is the negative role played by MIT in predicting control and relationships. Since correlation analyses showed positive coefficients, a possible explanation of the negative effects found in the regression analyses could be the existence of particular interactions between supervisors’ competencies and behaviours. A similar effect was detected in studies on safety leadership, where the so-called “inconsistent leadership” (i.e., leaders that display both safety-specific transformational leadership and passive leadership) predicted negative safety outcomes [44,45]. Similarly, even if supervisors’ competencies are all considered positive behaviours in the HSE framework, it is possible that, in some cases, they can interact negatively. For example, it is questionable whether certain behaviours included in the MIT competency (such as “Prefers to speak to me personally rather than use email”, “Socialises with the team” or “Takes an interest in my life outside work”) are desirable by employees when the supervisor is perceived as not respectful and honest and/or s/he doesn’t behave consistently and calmly (first competency). We suggest that future research on supervisors’ competencies should pay more attention to possible interactions existing between supervisors’ inconsistent behaviours that could determine negative effects.

### 4.1. Limitations

The research findings are mainly limited by the use of employees’ self-report questionnaires alone. As mentioned above, both employees and supervisors should fill in the SMCIT questionnaire in an upward feedback exercise. However, given the difficulty to obtain a high number of supervisors’ self-report questionnaires, we used only those of the employees for the evaluation of the psychometric properties of the 36-item SMCIT. Even if our results bring support to the validity and reliability of the employees’ questionnaire, the validity of the supervisors’ version of the SMCIT (i.e., the self-assessment) should also be evaluated.

A recent literature review on self–other rating agreement related to leadership in the workplace [46] showed that both self-ratings and others’ ratings are affected by various different factors. In particular, because ratings of leadership reflect not only the leader’s actual behaviour but also the rater’s cognitive schemas on effective leadership, specific cognitive processes could alter the rater’s evaluation. On the other hand, the authors [46] noted that ratings based on more behaviourally specific scales (as is the case of SMCIT) are less affected by the raters’ schemas and better reflect the real leadership behaviours.

In any case, the availability of wide samples of both employees and supervisors in future research could allow for the evaluation of the measurement invariance between the two groups, sustaining the comparison of the results in the upward feedback exercise.

### 4.2. Practical Implications

In order to ensure that the framework and the questionnaire were tailored to the user (i.e., line managers, Human Resource, and Occupational Health professionals, etc.), a usability analysis was conducted by the HSE [25]. Results showed that stakeholders felt the following: that the framework can be used in a stress management context; that the framework would be of use in a leadership development/training context; that the questionnaire could be used in a stress management context, as a next logical step after the HSE Stress Management Indicator Tool [25].

Indeed, the results obtained in this research have important practical implications. We believe that the availability of a valid and short 36-item SMCIT makes the analysis of supervisors’ competencies easier and more economically convenient for organizations. Thus, by stimulating this kind of practice two positive outcomes linked to work stress prevention can be achieved: the supervisors’ development and improvement of the psychosocial work environment; and the involvement of supervisors in the process.

First, as Kelloway and Barling [7] pointed out, supervisors’ development represents a truly innovative approach aimed to prevent and reduce stress at work through the improvement of the employees’ psychosocial work environment. Research conducted in the framework of the “Management Standards” [26] found that a better psychosocial work environment is not only linked to less negative consequences for the workers [47,48], but also to more positive outcomes, such as learning and performance [49,50,51]. Consequently, supervisors’ competencies development could be considered a more accentuated preventive strategy aimed not only at the reduction of work stress, but also the improvement of health, well-being, and motivation.

Second, supervisors are rarely fully involved in work stress prevention activities. However, they have been identified as the drivers of change [23], and many studies showed that on the basis of their attitudes and actions they can either “make or break” the intervention [52]. In fact, both in the UK [53] and the Italian context [54], it was found that the supervisors’ involvement represents an important enabling factor of the work stress prevention process. In this sense, the participation of supervisors in development activities could stimulate their involvement and the expected positive outcomes.

Finally, the availability of the short 36-item SMCIT (instead of 66) could make it easier to introduce other relevant variables in the questionnaire (e.g., stressors, coping strategies, well-being, etc.), stimulating further research and facilitating the development of knowledge in this important research area.

## 5. Conclusions

The study provides support to the validity and reliability of the short 36-item SMCIT. The brief version of the questionnaire represents an important step toward the development of interventions directed at supervisors in the work stress prevention process. Furthermore, we sustained the relations hypothesized between supervisors’ competencies and psychosocial factors, providing support to the criterion validity of the questionnaire and to the UK HSE “Management Competencies for Preventing and Reducing Stress at Work” framework.

## Figures and Tables

**Table 1:** Framework of the “Stress Management Competency Indicator Tool” (SMCIT) and hypothesized relations with the Management Standards and 36-item version.

	Sub-Competencies (in Brackets, the Relationship with Management Standards)	The 36-Item Version of the SMCIT (in Parentheses the Original Number in the 66-Item Version)
RR (17 items)	Integrity (Demands, Relationship)	1. Doesn’t speak about team members behind their backs (50)
		4. Is honest (51)
		7. Treats me with respect (53)
	Managing Emotions (Relationship)	2. Is consistent in his or her approach to managing (13)
		5. Acts calmly in pressured situations (45)
		8. Passes on his or her stress to me (46)
	Considerate approach (Control, Support, Relationship)	3. Creates unrealistic deadlines for delivery of work (4)
		6. Imposes ‘my way is the only way’ (20)
		9. Shows a lack of consideration for my work–life balance (36)
MCW (22 items)	Proactive work management (Demands, Support, Role)	10. When necessary, will stop additional work being passed on to me (2)
		13. Reviews processes to see if work can be improved (10)
		16. Prioritises future workloads (11)
	Problem solving (Demands, Support)	11. Follows up problems on my behalf (5)
		14. Is indecisive at decision-making (8)
		17. Deals with problems as soon as they arise (9)
	Participative (Demands, Control)	12. Gives me the right level of job responsibility (18)
		15. Encourages participation from the whole team (22)
		18. Correctly judges when to consult employees and when to make a decision (23)
RDS (12 items)	Managing conflict (Relationship)	19. Deals objectively with employee conflicts (37)
		22. Deals with employee conflicts head on (39)
		25. Acts as a mediator in conflict situations (43)
	Use of organizational resources (Support)	20. Seeks help from occupational health when necessary (64)
		23. Seeks advice from other managers when necessary (65)
		26. Uses HR as a resource to help deal with problems (66)
	Taking responsibility for resolving issues (Relationship)	21. Supports employees through incidents of abuse (38)
		24. Follows up conflicts after resolution (40)
		27. Makes it clear he or she will take ultimate responsibility if things go wrong (59)
MIT (15 items)	Personally accessible (Support)	28. Is available to talk to when needed (29)
		31. Returns my calls/emails promptly (30)
		34. Prefers to speak to me personally rather than use email (31)
	Sociable (Relationship)	29. Is willing to have a laugh at work (54)
		32. Socialises with the team (55)
		35. Brings in treats (56)
	Empathetic engagement (Control, Support, Relationship)	30. Takes an interest in my life outside work (61)
		33. Tries to see things from my point of view (62)
		36. Makes an effort to find out what motivates me at work (63)

RR = Respectful/Responsible; MCW = Managing and Communicating Work; RDS = Reasoning/Managing Difficult Situations; MIT = Managing Individual Within Team; HR = Human Resources.

**Table 2:** Means, standard deviations and Pearson’s correlation coefficients between the research variables (Cronbach’s alpha in parentheses).

	Mean	SD	1	2	3	4	5	6	7	8	9	10	11
1. RR	3.73	0.78	(0.87)										
2. MCW	3.56	0.79	0.78	(0.90)									
3. RDS	3.46	0.85	0.74	0.82	(0.92)								
4. MIT	3.54	0.84	0.71	0.81	0.78	(0.90)							
5. Demands	3.82	0.81	0.45	0.34	0.37	0.31	(0.81)						
6. Control	3.35	0.86	0.44	0.41	0.36	0.30	0.30	(0.85)					
7. Managers’ support	3.68	0.81	0.73	0.82	0.75	0.75	0.42	0.44	(0.84)				
8. Peer support	3.91	0.77	0.45	0.48	0.47	0.44	0.28	0.37	0.65	(0.86)			
9. Relationship	4.53	0.80	0.44	0.34	0.36	0.24	0.44	0.39	0.42	0.33	(0.82)		
10. Role	3.98	0.84	0.47	0.53	0.48	0.43	0.34	0.40	0.54	0.37	0.29	(0.83)	
11. Change	3.20	0.88	0.55	0.68	0.62	0.59	0.45	0.45	0.75	0.57	0.31	0.63	(0.78)

RR = Respectful/Responsible; MCW = Managing and Communicating Work; RDS = Reasoning/Managing Difficult Situations; MIT = Managing Individual Within Team. All correlations are significant at *p* < 0.001.

**Table 3:** Goodness of fit statistics (*N* = 353).

	χ^2^	df	CMIN/DF	Fit Indexes		
				CFI	RMSEA	SRMR
Model 1 (four factors)	1405.74	584	2.41	0.90	0.06	0.04
Model 2 (one factor)	1985.70	594	3.34	0.83	0.08	0.05

χ^2^ = Chi-square; df = degrees of freedom; CFI = Comparative Fit Index; RMSEA = Root Mean Square Error of Approximation; SRMR = Standardized Root Mean Residual.

**Table 4:** Multiple regression analyses predicting Management Standards.

Outcomes	Demands	Control	Managers’ Support	Peers’ Support	Relationship	Role	Change
Predictors							
RR	0.44 ***	0.32 ***	0.17 ***	0.15	0.46 ***	0.14	0.01
MCW	−0.08	0.29 **	0.43 ***	0.19 *	0.03	0.40***	0.49 ***
RDS	0.15	0.03	0.14 *	0.18 *	0.21 *	0.10	0.17 *
MIT	−0.06	−0.19 *	0.17 **	0.04	−0.28 **	−0.07	0.04
Overall R^2^	0.21 ***	0.22 ***	0.71 ***	0.26 ***	0.23 ***	0.30 ***	0.48 ***

Entries are standardized beta weights; RR = Respectful/Responsible; MCW = Managing and Communicating Work; RDS = Reasoning/Managing Difficult Situations; MIT = Managing Individual within Team; * *p* < 0.05; ** *p* < 0.01; *** *p* < 0.001.
